# Influencing factors and realization paths for smart community construction in China

**DOI:** 10.1371/journal.pone.0303687

**Published:** 2024-05-28

**Authors:** Wenjing Li, Lijian Zhang

**Affiliations:** 1 School of Public Policy and Administration, Chongqing University, Chongqing, China; 2 School of Marxism Studies, Chongqing University, Chongqing, China; Zhejiang Gongshang University, CHINA

## Abstract

The analysis of influencing factors serves as the cornerstone for the research on smart community construction. Drawing upon both field research and extensive literature study, this paper explores the influencing factors of China’s smart community construction and its effective paths by taking 52 national pilot zones for community governance and service innovation in China as examples. In the constructed analytical framework of influencing factors, elements such as economic development, capital investment, information infrastructure, community governance, public support system, and smart platform are included. By the use of the qualitative comparative analysis (QCA) method, the results of the study show that community governance, public support system, and smart platform are necessary conditions for smart community construction, while economic development, capital investment and information infrastructure play a leading role in the four combined paths. Finally, this study provides a new perspective for theoretical research, a reference forgovernmental departments to make decisions, and experience for the construction of smart communities in other developing countries.

## Introduction

In today’s digital era, smart community construction has become one of the important issues in many countries and regions. In 1992, the International Communication Center organization at the University of San Diego was the first to propose the concept of "smart communities" [1]. However, it did not attract enough attention at that time. Until, 2008 International Business Machines (IBM) Corporation put forward the concept of "Smart Earth" [2], and in 2009 further put forward the concept of smart city. Since then, the practice of smart community construction influence is increasing day by day. According to the United Nations, nearly 70 percent of the population is expected to live in cities by 2050 [3]. Smart cities provide conceptual and practical tools to deal this large-scale population movements. Multiple countries and regions launched smart city construction plans, such as the United States federal government’s "White House Smart City Action Initiative", Singapore’s "Intelligent Nation 2015", the European Union’s "Smart Cities and Communities Program", South Korea’s "Ubiquitous Network City" (U-City), and Japan’s "I-Japan Strategy" (I for Digital inclusion & innovation) [4]. In addition, many developing countries are actively engaged in construction smart cities. China launches "National Smart City Pilot" program. Brazil presents "My Smart City" project. India launches "Smart Cities Mission" (SCM) program. Vietnam has put forward the "National Action Plan for Digital Transformation". Thanks to smart city construction, Cape Town, South Africa, and Nairobi, Kenya are known as Africa’s City of Wisdom. Take China for example, as the world’s largest developing country, China in 2012–2022, the cumulative number of smart city pilots in China was approximately 800 [5], which is half the global number of pilots. This indicates that China is rapidly becoming the country with the largest smart city construction implementation worldwide.

Globally, the construction of smart communities has become a cutting-edge topic in urban development, with countries committing themselves to using information technology to enhance community governance, optimize public services and improve the quality of life of residents [6]. Smart community refers to the complete implementation of information technology applications, such as big data, cloud computing, and artificial intelligence, integrating all types of service resources in the community and creating a new form of community governance based on informatization (informatization is the use of electronic information technology to achieve a high degree of sharing of information resources, explore the potential of social intelligence, and promote economic and social development) and intelligent management and services [7].

However, we are facing a series of challenges and issues in theoretical research and practice, the solution of which is crucial. In terms of theoretical research in developed countries for examples, the researchers focuses on the analysis of the problems and paths of smart community construction, and not on the analysis of influencing factors. Considering the practical level, many communities still face the issues such as imperfect technical facilities and unstandardized data management. In addition, community resident, understanding of and participation in the construction of smart communities is not high due to lack of effective community governance and resident participation mechanisms, limited attention to social aspects [8], which results in difficult for widely reorganization and supportation of the construction of smart communities, leading to prevents it from giving full play to its potential.

Because it is not easy to realize smart communities. we need to consider complexity and diversity of factors that influence them.

In this context, we further consider: what are the factors affecting the construction of smart communities? What are effective paths to construction smart communities? This not only involves factors such as technical, economic and policy and other factors, but also the need to take into account the impact of community residents’ participation, cultural traditions and geographical characteristics. Therefore, we need to reveal the intrinsic connections and interactions among these factors through in-depth research and analysis.

In order to answer these questions, this study will adopt the qualitative comparative analysis (QCA) method and take 52 of National Pilot Zones for Community Governance and Service Innovation in China as the research object to deeply in depth the influencing factors of China’s smart community construction. Through the systematic study of these factors, we expect to provide theoretical support and practical guidance for the construction of smart communities and to promote the innovation and development of community governance and services. The significance of this study is as follows: First, considering China as an example, this study identifies the factors influencing smart community construction, clarifies the important roles played by these factors in smart community construction, and provides useful information on the practice of smart community construction in developing countries. Second, the use of the QCA-based methodology enables an examination of the influencing factors and construction paths of smart community construction and helps expand the research perspective of smart community construction. Third, based on QCA results, we put forward some valuable suggestions to China and other developing countries on effectively promoting smart community construction.

### Literature review

Smart communities have emerged as a focal point in contemporary urban development strategies, garnering significant attention as an innovative approach to national governance and urban revitalization. In numerous countries, the construction of smart communities has become integral to overarching strategies aimed at transforming and upgrading urban landscapes. Presently, scholarly discourse on factors influencing the construction of smart communities predominantly revolves around three key dimensions: concept delineation [6], primary stakeholders, and efficacy evaluation [8].

First, conceptual clarity serves as the cornerstone of research into smart community construction. Across scholarly works, the definition and interpretation of smart communities vary considerably. Caragliu, Del Bo and Nijkamp (2011) proposed a conceptual framework for smart city, characterizing them as an urban entities harnessing information and communication technology to bolster urban management efficiency and enhance residents’ quality of life [4]. John M Eger (2011)views a "smart community" as a service model seamlessly integrating residents needs into cyberspace [9], offering a more efficient approach to societal management [10]. Central to the concept of Smart communities is the pivotal role of information technology in bolstering community governance and services [11–15]. Attributes of smart communities, encompass smart infrastructure, information-driven management, active community participation [8] and intelligent, informative and ecological [16]. Moreover, these smart communities fulfill multifaceted roles, during the spread of COVID-19, there was a significant reduction in the chance of population cross-infection [17–20], and offering residents a diverse array of smart services [21], such as a smart home, smart healthcare, smart security, and a smart government [22]. Based on this, many scholars have explored related fields such as smart transportation [23], such as electric mobility (eMobility) [24], and smart grid [25] etc, which provide useful references for us to carry out research on smart community construction.

Second, the influencing factors of smart community construction are explored from the perspective of its primary stakeholders. At the policy level, the pivotal role of the Communist Party of China (CPC) in fostering smart communities is widely acknowledged [26–30]. Governmental policy formulation and resource allocation stand out as decisive factors in driving smart community development [31, 32]. Moreover, the engagement and support of social organizations [33–35], alongside active participation from community residents, emerge as critical catalysts for advancing smart community initiativesand [36–39]. Concurrently, technological innovation plays an instrumental role in propelling smart community construction forward [1].

Third, Scholars have also achieved certain results in terms of specific effectiveness evaluation and empirical research. Smart communities are key construction subprojects of smart cities [40], the researches related to smart cities is also applicable to smart communities to a certain extent. Regarding the dimensions of smart cities, scholars have proposed various dimensions, encompassing economic, smart governance, smart people, smart environment, smart mobility, and smart living [41], or management and organization, technological infrastructure, local governance, policy context, people and communities, local economy, built infrastructure, and natural environment [42]. Study shows that public services and facilities, physical and environmental well-being, and a sense of community play pivotal roles in shaping smart city evaluation frameworks [43]. Specific indicators include, infrastructure, policy, governance structure, organizational management, economy, technology, and people and communities [44]. For instance, studies in India have focused on demographic dynamics, economic indicators, infrastructure development, and quality of life metrics [45]. Analyzing initiatives such as the "100 Smart Cities Mission," researchers have underscored dimensions such as ease of living, smart governance, connected communities and urban resilience [46]. In regions like Curitiba, Brazil, researchers have identified key determinants of quality of life in smart communities, emphasizing socio-structural relationships, environmental stewardship, material well-being, and community integration [47]. Studies in Brazil’s major smart cities, such as Rio de Janeiro, Porto Alegre, and Belo Horizonte, have highlighted the pivotal role of information and communications technology (ICT) in enhancing governance effectiveness and stakeholder integration [48]. A study of Brazilian smart cities uses Gross Domestic Product (GDP) to measure the level of governance of a city and argues that technology is a key factor in the development of a smart city, using the number of households with computer to measure [49]. A study of 10 smart cities in Vietnam shows that smart infrastructure, smart human capital development, smart economy, smart government, and smart environment are the key factors influencing their development [50]. In addition, empirical studies examining smart city projects, such as the Trento Smart City initiative in Italy, offer insights into strategic drivers and decision-making processes crucial for sustainable smart community development [51].

The evaluation of smart communities has been approached from various perspectives in scholarly research. The Intelligent Community Forum, for instance, has scrutinized aspects such as broadband connectivity, knowledge-based workforce, innovation, digital inclusion, marketing, and advocacy efforts [52]. Moreover, researchers have proposed quality criteria for intelligent communities, including fine-grained service perception, interactive information platforms, intelligent public services, and collaboration networked actions [53], and advocate for comprehensive evaluation frameworks, including guarantee (includes four second-level indexes of policies and regulations, design guarantee, operation guarantee and other guarantees), information infrastructure, municipal infrastructure, platform, application, and evaluation layers [54]. Niaros et al (2017) proposed a multidimensional and multilevel study encompassing government-level support, socio-economic needs, dimensions of the built environment, and levels of connected transportation [55]. Dunn (2002) developed a comprehensive evaluation system for smart communities focusing on effectiveness, efficiency, economy, and equity [56].

Furthermore, in empirical researcher, various methodologies have been employed. Structural equation modeling (SEM) has been utilized to assess smart communities across dimensions such as eco consciousness, education, creativity, digital proficiency, and social cohesion [17]. For instance, one researcher constructed a model using the Criteria Importance Though Intercrieria Correlation (CRITIC)-Technique for Order Preference by Similarity to an Ideal Solution (TOPSIS) method empirically analyzing smart communities in multiple Chinese cities [57]. Additionally, studies have evaluated the sustainability of smart communities in terms of their guarantee system, infrastructure, community services, and community management [40]. Another study identified factors affecting smart community in Beijing, including community hardware, network facilities, system platforms, technology level, resource integration, personnel quality, investment funds, and community residents’ awareness levels [58]. Some scholars have devised comprehensive evaluation frameworks for residents’ sense of gain in smart communities, integrating methodologies such as the entropy weight method (EWM), fuzzy comprehensive evaluation (FCE), and obstacle degree model (ODM) [59]. Moreover, comparative study of 42 Chinese urban communities is conducted by using the fuzzy set qualitative comparative analysis (fsQCA) method, drawing on the theory of value co-creation. The results show that government bodies and social organizations are the core conditions for the realization of smart community informatization [60].

In summary, despite the plethora of studies on smart cities and smart communities studies in developed countries, there remains a relative dearth of research focused on developing countries [47, 52]. Systematic investigations into the factors influencing the construction of smart communities have garnered limited academic attention thus far. Existing has predominantly centered on qualitative analysis or the development of evaluation indices, often overlooking the nonlinear relationship and diverse arrayof influencing factors. This limition constrains our comprehensive understanding of smart community construction processes. Existing research tends to focus on communities or cities as the object of study, and lacks research on multiple smart community construction pilot zones covering the whole country, making research on deeper and more comprehensive influencing factors affecting the construction of smart communities somewhat lacking. Therefore, this paper takes 52 National Pilot Zones for Community Governance and Service Innovation in China as an example, constructs an analytical model of influencing factors for smart community construction, and conducts an empirical study using fuzzy set qualitative comparative analysis (fsQCA) in order to derive the key factors and multiple combinations of paths influencing the construction of smart communities.

## Methods and materials

### Introduction to Qualitative Comparative Analysis (QCA)

The emergence and development of QCA: the method of QCA finds its roots in the social sciences, and its inception dating back to the 1980s. Development by the American sociologist Charles C. Ragin [61]. QCA offers a case-study oriented approach for examining a collection of theories, aimed at uncovering how multiple factors collaboratively influence a specific outcome. QCA emphasizing non-linear causality, multifactorial interactions, and complexity analysis, provides a comprehensive framework for analyzing intricate relationships. QCA encompasses three s primary operational methods: crisp set (csQCA), fuzzy set (fsQCA) and multi value set (mvQCA). At its core, QCA draws upon Boolean algebra and mediation theory. Boolean algebra, a fundamental component of QCA facilitates the processing and analysis of relationships between conditions and outcomes through logical operators (AND, OR, and NOT). By integrating elements of both qualitative and quantitative research, QCA allows for identification of patterns and key factors through systematic comparisons across multiple cases. Over time, QCA has evolved into versatile research methodology applicable across various disciplinary domains, including political science, sociology, management, public policy, and environmental studies.

Basic analysis logic of QCA. First, differences from quantitative analysis: In quantitative analysis, statistical methods are often used to explore relationships between variables and to quantify the strength and direction of those relationships. QCA, on the other hand, is different in that it focuses more on explaining the occurrence of particular outcomes, emphasizing the complexity and diversity of causal relationships rather than simple numerical relationships. Unlike the linear causality analysis logic of quantitative research, QCA asserts that the influence of conditional variables on outcome variables is not singular, independent, or linear. Instead, it emphasizes that the occurrence of a particular social phenomenon can be attributed to the combination of multiple factors [62]. It is concerned with the presence or absence of various combinations of factors, rather than just the strength of causal relationships. Second, the unit of analysis is a combination of conditions rather than a case: in QCA, the unit of analysis is a combination of conditions rather than a single case. QCA treats different combinations of conditions as paths leading to a particular outcome, and understands the mechanism of outcome formation by comparing the commonalities and differences between these paths. Third, inferential logic based on necessary and sufficient conditions: QCA is based on Boolean logic and emphasizes the relationship between necessary and sufficient conditions. Necessary conditions refer to conditions that must exist in a given situation, while sufficient conditions refer to conditions that are sufficient to lead to a particular outcome. By analyzing combinations of these conditions, QCA is able to identify which conditions are necessary and which are sufficient, as well as the relationship between them.

The rationale for selecting the QCA methodology is as follows: First, the National Pilot Zone for Community Governance and Service Innovation is a complex system comprising multiple factors, such as policy support, technological foundation, community governance mechanisms, and citizen participation. These factors determine the system’s success or failure. It is difficult to capture such multiple influences using traditional single-factor analysis.

Second, QCA emphasizes nonlinear causality, that is, the interrelationships and complementarities among conditional variables. In the National Pilot Zone for Community Governance and Service Innovation, factors may interact in different ways, which results in different outcomes, QCA helps identify these nonlinear relationships.

Third, QCA involves the comparison of multiple cases to provide an in-depth clarification of the combinations of factors in multiple contexts. Hence, QCA can overcome the disadvantages of case and univariate studies. The comparative analysis of multiple cases helps identify the causes of events and clarify the possible combinations and interactions of multiple influencing factors. The effect generation of smart community construction, which is a systematic engineering process, is influenced by combinations of multiple internal and external factors. Moreover, QCA can accurately extract the key factors affecting smart community construction and, thereby, help analyze the combination of and interactive relationships among the factors affecting the construction.

Finally, the number of national pilot zone for community governance and service innovations is 114. This is consistent with one of the strengths of QCA, that is, its suitability for analyses involving small to medium sample sizes. In this study, 52 subjects were selected from the 114 pilot zone, which is a small to medium sample size.

Therefore, QCA is suitable for analyzing the national pilot zone for community governance and service innovation.

### Sample selection

In order to promote community governance and service innovation and promote the construction of smart communities, the Ministry of Civil Affairs (MCA) established four batches of a cumulative total of 114 national pilot zone for community governance and service innovation in 2011–2019 [63], and each pilot zone carried out creation activities around the experimental themes and tasks. We selected 52 pilot zones as the subjects of this paper based on the following three principles. First, representativity: pilot areas with different characteristics are selected, covering different geographic regions, levels of economic development, population size, industry structure and other factors, and also including pilot zones in the first, second, third and fourth batches, and the pilot areas have clear goals and tasks for smart community construction, in order to comprehensively analyze the influencing factors of smart community construction in the country from multiple perspectives. Second, typicality: We select typical pilot areas as samples, which have achieved certain results in the construction of smart communities and have passed the acceptance of the Ministry of Civil Affairs (MCA), all the aspects of their construction and operation are relatively mature, forming a certain demonstration effect and providing valuable cases and experiences. Third, operability: Considering the operability of the actual investigation, pilot districts that can obtain data and information relatively easily are selected as samples, such as pilot district construction programs, summary reports, etc., to ensure that the study can be carried out effectively.

The steps of case selection are as follows: firstly, the total of 114 cases of all batches of National Pilot Zones for Community Governance and Service Innovation are taken as the first-level alternative case base; secondly, the typical case base is screened and determined according to the three principles set in this study; finally, the supporting materials of the cases (Implementation plan of the pilot zone, summary report, news report, etc.) are searched, and sorted out, and the cases are repeated and confirmed through the triangulation method, so as to form the final study case base required for the present study.

Based on above principles and steps, a total of 52 pilot zones were selected for this study (See Table 1). These include 5 pilot zones in the first batch, 12 in the second batch, 22 in third batch, and 13 in the fourth batch. Regarding regional distribution, these pilot zones are spread across the country, including 28 in the eastern region, 14 in the central region, and 10 in the western region. However, regarding the nature of cities, the selected pilot zones include 4 prefecture-level cities, 45 precincts of cities, and 3 county-level cities. These factors indicate that the sample selection is scientific and comprehensive. For the location distribution of the pilot zones, please see Table 1.

**Table 1:** 52 sample national pilot zones for community governance and service innovation.

Batch	Pilot Zones
First (5 samples)	Shenhe District, Shenyang, Liaoning Province;Baita District, Liaoyang, Liaoning Province;Wuxi, Jiangsu Province;Hangzhou, Zhejiang Province;Haicang District, Xiamen, Fujian Province.
Second (12 samples)	Chaoyang District, Beijing;Qingshan District, Baotou City, Inner Mongolia Autonomous Region;Erlianhot, Inner Mongolia Autonomous Region;Shahekou District, Dalian, Liaoning Province;Nangang District, Harbin, Heilongjiang Province;Xuhui District, Shanghai;Suzhou Industrial Park, Jiangsu Province;Huangdao District, Qingdao, Shandong Province;Kaifu District, Changsha, Hunan Province;Yuexiu District,Guangzhou, Guangdong Province;Chenghua District, Chengdu, Sichuan Province;Panlong District, Kunming, Yunnan Province.
Third (22 samples)	Xicheng District, Beijing;Haidian district, Beijing;Hexi District, Tianjin;Guangyang District, Langfang, Hebei Province;Hongshan District, Chifeng, Inner Mongolia Autonomous Region;Kuancheng District, Changchun, Jilin Province;Longsha District, Qiqihar, Heilongjiang Province;Jianye District, Nanjing, Jiangsu Province;Gulou District, Nanjing, Jiangsu Province;Yunlong District, Xuzhou, Jiangsu ProvinceGusu District, Suzhou, Jiangsu Province;Shangcheng District, Hangzhou, Zhejiang Province;Lixia District, Jinan, Shandong Province;Shibei District, Qingdao, Shandong Province;Taishan District, Tai’an, Shandong Province;Jianxi District, Luoyang, Henan Province;Zhijiang City, Yichang, Hubei Province;Qingyang District, Chengdu, Sichuan Province;Xixiu District, Anshun, Guizhou Province;Qujing Qilin District, Yunnan Province;Beilin District, Xi’an, Shaanxi Province;Jintai District, Baoji, Shaanxi Province.
Fourth (13 samples)	Shijingshan District, Beijing;Changning District, Shanghai;Gulou District, Xuzhou, Jiangsu Province;Yuecheng District, Shaoxing, Zhejiang Province;Quzhou City, Zhejiang Province;Baohu District, Hefei, Anhui Province;Yidu City, Hubei Province;Xianning City, Hubei Province;Chancheng District, Foshan, Guangdong Province;Jiangbei District, Chongqing; Jinniu District, Chengdu, Sichuan Province;Xindu District, Chengdu, Sichuan Province;Suzhou District, Jiuquan, Gansu Province.

### Data

In this study, the main data sources were the results compiled and published by Ministry of Civil Affairs (MCA) on the construction of first and second pilot zone batches, such as the Innovative Practices in the Perspective of Community Governance Modernization: A Compilation of achievements the First Batch of National Pilot Zone for Community Governance and Service innovation [64] and Footsteps of innovation: A collection of achievements of national pilot zone for community governance and service innovation [65]. In addition, research data were obtained from some books, such as The China urban community governance report (2018) [66] and Community Consruction with Chinese Characteristics Shenyang Shenhe District Experience [67]. Furthermore, several valuable authoritative sources, including the official website of the Ministry of Civil Affairs (MCA), People’s Daily Online, the official website of the Xinhua News Agency, the official websites of local governments, statistical yearbooks, the construction plans of pilot zones, and governmental work reports, were consulted.

### Research design

#### Establishment of research variables

According to established research methodologies, it is generally recommended to employ between four to seven conditional variables when dealing with medium-sized samples,typically ranging from 40–80 cases [68]. In alignment with this principle, our study, delves into the impact of smart community construction by considering four dimensions: central promotion, social affirmation, local recognition, and residents’ satisfaction. Furthermore, we identify six key dimensions that play pivotal roles in influencing smart community construction. These dimensions include economic development, capital investment, information infrastructure, community governance, public support system, and smart platform. The comprehensive nature of these variables is outlined in Tables 2 and 3.

**Table 2:** Measurements and sources of outcome variables.

Outcome variable	Construction effect	Standard measure	Literature source
Central promotion	Generating replicable innovations	Selected as one of the "Top Ten Innovative Achievements in Community Governance in China"/"100 Excellent Community Work Methods in China"/"Typical Cases of Grassroots Governance Innovation in China"	[61–63]
Social affirmation	Centralized media reports	Positive coverage in People’s Daily (People’s Daily Online) or the official website of the Xinhua News Agency	[51, 61, 62]
Local recognition	Consolidation of Creation Achievements	Governmental work report mentions the consolidation of the results of pilot zone creation	[63]
Residents’ satisfaction	Intelligent applications	Realizing smart government, smart elderly, and smart life	[39, 60]

**Table 3:** Measurements and sources of conditional variables.

Conditional variable	Influential factor	Standard measure	Literature source
Economic development (V1)	Level of urban economic development	Whether it is a first- or second-tier city Whether the Gross Domestic Product (GDP) per capita is average or not	[38, 41, 42, 49, 58, 63]
Capital investment (V2)	Government’s financial inputs	Increase in expenditure on urban and rural community affairs	[42, 44, 58, 77]
	Social capital inputs	Whether to establish a diversified social finance participation mechanism or not	
Information infrastructure (V3)	Status of network-connected devices	Whether there is an increase in the number of computers per 100 urban residents or not	[43, 44, 49, 52, 54]
	Mobile telephone penetration	Whether the number of cell phones per 100 urban residents increased or not	[38, 77]
	Internet penetration	Whether the number of Internet broadband access subscribers increased or not	
Community governance (V4)	Organizational leadership	Whether to set up a working group to supervise the experimental zone headed by the main leader of the district committee and district government or not	[27–30, 33, 70]
	Community party building	Whether to promote community (regionalized) party building or not	[9, 34–38]
	Coordinated governance	Whether social organizations are deeply involved in community governance	
	Community autonomy	Community residents’ participation in consultation and decision-making	
Public support system(V5)	Policy support	Availability of supporting policies	[11, 15]
	Institutional support	Whether to promote street system reform or not	[54, 75]
	Talent support	Whether to strengthen gridded personnel management or not	
Smart platform(V6)	Management platform	Establishment of a smart management system or improvement of a comprehensive information platform for community public services	[43, 44, 52, 54, 73, 74]
	Service platform	Launching a community hotline, website, or WeChat public number	[53, 54, 58]

#### Measurement of research variables

In this study, based on the theory of community governance and the construction of smart communities in the experimental area, a four-valued fuzzy set (0, 0.33, 0.67, 1) was used to measure the research variables corresponding to "completely unaffiliated, partially unaffiliated, partially affiliated, completely affiliated". The rationale for this choice is that the experimental area’s case information is relatively detailed and includes background information, specific practices and their effectiveness, and expert comments; moreover, the construction effect cannot be distinguished by dichotomy alone. Therefore, this study retained detailed information about the cases and used four-valued fuzzy sets to accurately reflect the construction effect.

### Variable coding rules

#### Outcome variable coding rules

The outcome variable (Y) reflects the impact of smart community construction and encompasses four dimensions: central promotion, local recognition, social affirmation, and residents’ satisfaction.

Central promotion. This indicator assesses whether the pilot zone had generated innovations that can be be widely disseminated and adopted in other communities. It is gauged based on whether the pilot zone has been recognized as one of the "Top Ten Innovations in Community Governance in China," [63, 69], "100 Excellent Community Work Methods in China," [70] and "Typical Cases of Grassroots Governance Innovation in China." [71].

Social affirmation. China’s major media, such as People’s Daily, People’s Daily Online, and the official website of the Xinhua News Agency, serve as barometers to gauge whether the construction of pilot zone has demonstrated positive effects at the social level. Social affirmation is determined by assessing whether the media has reported and positively publicized the construction of pilot zone.

Local recognition. This dimension focuses on the sustainability of pilot zone construction, specifically,, whether the local government is inclined to further promote the construction of pilot zones after their initial acceptance. This is measured by whether the local government’s work report mentions "consolidating" or "deepening" the the results achieved in pilot zones.

Residents’ satisfaction. Community satisfactionserves as a key evaluation criterion for residents regarding community service andreflects the development status of a smart community [39]. Residents’ satisfaction hinges on their experiences with smart applications, such as smart government, smart aging, and smart living. This dimension is assessed based on the effective delivery of these smart services.

Coding Rules: Completely affiliated is coded as 1, partially affiliated as 0.67, partially unaffiliated as 0.33, completely unaffiliated as 0.

#### Conditional variables coding rules

Economic development (V1). The economic vitality of a city stands as a pivotal determinant influencing the trajectory of smart community construction. In our analysis, city classification and per capita Gross Domestic Product (GDP) serve as yardsticks for assessing local economic development [4, 63]. This dimension is gauged by factors such as whether the city belongs to the first-tier or second-tier category, or if its GDP per capita surpasses the municipal (provincial) level threshold. If completely affiliated coded 1, partially affiliated coded 0.67, partially unaffiliated coded 0.33, completely unaffiliated coded 0.

Capital investment (V2). Government’s financial allocations constitute the primary source of funding for smart community construction, although supplementary, social funds are also indispensable for the advancement of these initiatives. We measure by this variable by tracking the increment in urban and rural community affairs expenditure and establishing a mechanism to encourage diverse social fund participation. If completely affiliated coded 1, partially affiliated coded 0.67, partially unaffiliated coded 0.33, completely unaffiliated coded 0.

Information infrastructure (V3). The success of smart community construction hinges significantly on the judicious selection of appropriate information infrastructure [72, 73]. In our investigation, we evaluate information infrastructure across three dimensions: network connectivity, cell phone penetration, and internet penetration. Specifically, we gauge growth in the number of computers per 100 urban residents, the prevalence of cell phones per 100 urban residents, and and the expansion of internet broadband access subscribers as the standard metrics. If completely affiliated coded 1, partially affiliated coded 0.67, partially unaffiliated coded 0.33, completely unaffiliated coded 0.

Community governance (V4). This variable encompasses the establishment of an efficient community governance framework, which entails organizational leadership, community party building, collaborative governance, and community self-governance. Measurement criteria include the establishment of aleadership working group headed within the pilot area, spearheaded by the main leader of the district, the promotion community (regionalized) party building, facilitation of social organizations, and ensuring active participation of community residents in decision-making processes. If completely affiliated coded 1, partially affiliated coded 0.67, partially unaffiliated coded 0.33, completely unaffiliated coded 0.

Public support system (V5). Institutions (governance systems, policies, and regulations), individuals, and financial resources collectively play pivotal roles in facilitating the implementation of smart governance of urban communities [74, 75]. The "Guide to the Construction of Smart Communities (Trial)" highlights the significance of "policy guarantee (formulation of policies for the construction and operation of smart communities)" and "financial guarantee (funding planning and safeguards for smart community construction)". as essential control elements, imperative for the successful execution of smart community construction. Consequently, our measurement criteria encompass the assessment of whether policies supporting pilot zone construction have been, introduced, the execution of reform within the street system, and enhancement of grid personnel management. If completely affiliated coded 1, partially affiliated coded 0.67, partially unaffiliated coded 0.33, completely unaffiliated coded 0.

Smart platform (V6). At the core of smart community construction lies the establishment of smart platforms, including but not limited to a smart community management system, a comprehensive information platform forpublic services within the community, and an online new media service platform. Evaluation of this dimension revolves around the construction and effective operation of management and service platforms. If completely affiliated coded 1, partially affiliated coded 0.67, partially unaffiliated coded 0.33, completely unaffiliated coded 0.

#### Example of a test area and fuzzy set assignment table

This section details an example of a pilot zone. Based on the variable setting and coding rules mentioned in the preceding section and an in-depth analysis of relevant research materials in the pilot zone, the original materials and coding table for the test area depicted in Table 4 were formed.

**Table 4:** Examples of a pilot zone.

Variable		First batch (trial period: 2011–2015) Location: Wuxi, Jiangsu Province	QCA code
Outcome variable	Centralized promotion	No community governance awards	0.33
	Local recognition	The 2016 governmental work report did not mention the consolidation of the results of pilot zone construction	
	Social affirmation	People’s Daily Online reports: Jiangsu Wuxi 1 + 2 model of innovative community management	
	Residents’ satisfaction	The Wuxi City Community Management Services Comprehensive Information Platform integrates residential affairs and governmental affairs and services. For example, the Meihu community established the WeChat public number "Meihu Community Workstation," which places community dynamics, clerical guidelines, property management, the surrounding business circle, and other community services on WeChat	
Economic development	Level of urban economic development	Wuxi is a second-tier city	0.67
Capital investment	Government’s financial investment	Expenditures on urban and rural community affairs: $100,308,400,000 in 2012; $1,146,226,000 in 2013, with an increase in inputs	1
	Social capital investment	The Community Intelligent Information Screen project is led by the Office of the Leading Group of Informatization of Wuxi Municipality, which integrates and provides information resources, and is co-financed and operated by China Telecom Wuxi Branch and DEYA Intelligent Technology Wuxi Co.	
Information infrastructure	Status of network-connected devices	Computers per 100 urban residents: 215.4 in 2012; 221.3 in 2013. An increase in numbers	0.67
	Mobile telephone penetration	Ownership of mobile telephones per 100 urban residents: 112.1 in 2012; 105.1 in 2013. A decrease in numbers	
	Internet penetration	Number of Internet broadband access subscribers: 1.41 million in 2012; 1.44 million in 2013. An increase in volume is shown	
Community governance	Organizational leadership	The establishment of a group leading harmonious urban–rural community construction, the district party committee, and the main leaders of the district government as the group leaders	1
	Community party building	Building a new type of grassroots organization having grassroots party committees as its core and various types of grassroots groups as the main body	
	Coordinated governance	Promoting community service socialization. At the municipal and district levels, numerous policy documents have been issued to promote the development of social organizations, special funds have been arranged to support the construction of social organization incubation bases at all levels in various regions, and the practice of generating public welfare venture capital has been carried out	
	Community autonomy	Democratization of residents’ self-governance. The city’s neighborhood committees have generally established 12 democratic systems, with a 99% self-control rate. A community consultation model has been developed, with an "open space" as the main form	
Public support system	Policy support	Twelve policy documents, including the Implementation Plan for Construction a National Pilot Zone for Community Management and Service Innovation and the Opinions on Strengthening Community Management and Service Innovation, have been issued	1
	Institutional support	Flat community management has been implemented to enable the complete coverage of the city’s "one committee, one residence, one station, one office" strategy	
	Talent support	Every 300 households are divided into a chartered area, and one social work station staff member is made responsible for the chartered area	
Smart platform	Management platform	The establishment of a comprehensive information platform for community management services in Wuxi City, which integrates residential and governmental affairs and services. Some communities have established WeChat public numbers for smart communities, as well	1
	Service platform	Establishment of the Wuxi Citizen Service Center, which relies on the 24-hour, all-weather 96158 municipal citizen service call platform, various citizen service websites, and community service stations to provide the citizens of Wuxi with citizen services, such as informatized home-based elderly care services, family life services, and livelihood commodity delivery services, using information technology	

Note: Due to space limitations, this study describes the source material and coding for only one case. For a full list of case codes, please see the Supporting information of this paper (S1 Datasheet).

#### Fuzzy set assignment table for pilot zone

According to the assignment rules discussed in earlier sections, we coded all 52 Pilot Zone and depicted the results in the assignment table shown in Table 5.

**Table 5:** Fuzzy set assignment table for pilot zone.

S/ N	Pilot Zone	Y	Conditional variable					
			V1	V2	V3	V4	V5	V6
3	Wuxi City, Jiangsu Province	0.67	0.67	1	0.67	1	1	1
11	Xuhui District, Shanghai	0.33	1	1	0.33	1	1	1
17	Panlong District, Kunming City	1	0.33	0.67	1	0.67	0.67	0.67
20	Hexi District, Tianjin	1	1	0.67	0.67	1	0.67	1
36	Xixiu District, Anshun City	0.67	0.33	1	0.67	0.67	1	1
52	Suzhou District, Jiuquan City	0.33	0	0.33	0.67	1	0.67	1

Note: Y = outcome variable; V1 = economic development; V2 = capital investment; V3 = information infrastructure; V4 = community governance; V5 = public support system; V6 = smart platform. Due to space limitations, the table presents only some cases.

## Empirical analysis

The necessity analysis of univariate conditional variables was performed. In Boolean algebraic logic, a necessity condition indicates that when an outcome occurs the conditional variable must occur, and the necessity condition adequately explains a causal path leading to a certain outcome. In the necessity analysis conducted in this study, Consistency and Coverage were the core measures of necessity conditions. Among the two, the Consistency metric is particularly important. Consistency is the degree to which the empirical data of the selected case matches the expected relationship between the condition variable and the outcome variable, and its numerical magnitude allows the determination of the necessary and sufficient relationship of the condition variable to the outcome variable. The consistency equation as follows (see Eq 1).


$$\text{Consistency}\left(\text{Xi}\leq \text{Yi}\right)=\sum \left[\text{min}\left(\text{Xi},\;\text{Yi}\right)\right]/\sum \text{Xi}$$ (1)

The value of coverage indicates the explanatory power of the condition variable for the outcome, with higher values indicating that the causal variable explains a higher proportion of cases. The coverage equation as follows (see Eq 2).


$$\text{Coverage}\left(\text{Xi}\leq \text{Yi}\right)=\sum \left[\text{min}\left(\text{Xi},\;\text{Yi}\right)\right]/\sum \left(\text{Yi}\right)$$ (2)

Xi (set of combinations of conditional variables): this denotes a set of combinations of conditional variables, which can be a set of one or more conditions, which are considered to influence a certain outcome in a certain case.

Yi (the set of case outcomes when they occur): this is the set to which the corresponding case belongs when a certain outcome occurs. This set may be the set of cases that have a certain characteristic, exhibit a certain behavior, or achieve a certain state.

In QCA, Xi and Yi are analyzed comparatively to understand the extent to which the combination of conditioning variables (Xi) affects the case outcome (Yi), i.e., the extent to which Xi is a sufficient condition for Yi (consistency) and the extent to which Xi is a necessary condition for Yi (coverage).

In QCA, the Consistency metric measures the degree of consistency between the conditional and outcome variables. When a conditional variable’s consistency value is greater than or equal to 0.9, it can be considered a necessary condition for the outcome variable. A consistency value above 0.8 is the fulfillment criterion for the sufficient condition. In addition, the coverage indicator measures the condition’s explanatory power; the higher the coverage value, the stronger the explanatory power.

Next, we import Table 5 into fsQCA3.0 software, select "Necessary conditions" in "Analyze", and select the corresponding condition variables and outcome variables in "Add conditon" and "Outcome" respectively, finally get the results in Table 6.

**Table 6:** Necessity analysis of individual variables.

Conditional variable	Y = high level of smart community construction effectiveness		~Y = non-high level of smart community construction effectiveness	
	Consistency value	Coverage value	Consistency value	Coverage value
V1	0.748124	0.682307	0.818409	0.620274
~V1	0.605573	0.795401	0.593919	0.669169
V2	0.855663	0.691197	0.844231	0.584993
~V2	0.486245	0.784438	0.554352	0.767147
V3	0.808146	0.678261	0.845481	0.608696
~V3	0.533762	0.801072	0.553103	0.712064
V4	0.964273	0.594624	1.000000	0.528971
~V4	0.236156	1.000000	0.233653	0.848714
V5	0.952483	0.648820	0.985839	0.576053
~V5	0.377635	0.968836	0.399000	0.878093
V6	0.988210	0.567967	1.000000	0.493019
~V6	0.117899	1.000000	0.123698	0.900000

Note: V1 = economic development; V2 = capital investment; V3 = information infrastructure; V4 = community governance; V5 = public support system; V6 = smart platform. The symbol "~" is the Boolean algebra symbol for "not."

Table 6 demonstrates high consistencies among community governance (V4), public support system (V5), and smart platform (V6) each exceeding 0.9. This indicates that these three conditional variables as necessary conditions for successful smart community construction.

This finding aligns with research conducted in developing countries such as China, Vietnam, and India, where successful smart community initiatives emphasize the pivotal role of community governance. The transformation of government attitudes towards transparency and resource-sharing is essential in fostering successful smart community endeavors. Additionally, an effective policy framework, coupled with the establishment of versatile smart platforms facilitating interaction and service provision, are key components of successful smart community implementation [43, 45, 76–78]. Studies, such as the one conducted in Aarhus, Denmark in 2016, underscore the importance of collaborative efforts among citizens, businesses, knowledge institutions, and municipal agencies in smart community development [79]. Government entities and social organizations as highlighted by Zhangfan and Cao Zhili, play crucial roles in smart community construction. Street offices act as facilitators, aligning higher level government policies with local realities for grassroots implementation. Furthermore, community self-governance is through the establishment of community party organizations and residents’ self-governance organizations, with party organizations play assuming role in ideological leadership, political protection and spiritual cohesion [60]. However, the consistency of other conditional variables is lower than 0.9; therefore, these variables do not satisfy the criterion of necessary conditions for smart community construction.

### Truth table construction

In the fsQCA3.0 software operation, we imported Table 5 data and set the frequency and consistency thresholds for the research cases. The frequency threshold is related to the number of study samples. For small study samples, the threshold can be set to 1 [80]. Regarding consistency threshold, Ragin and Fiss suggests setting it to no less than 0.75 and proposes using a threshold value of 0.8 or higher [68, 81] Therefore, in this study, we set the frequency threshold to 1, raw consistency threshold to 0.8, and PRI (Proportional Reduction in Inconsistency) consistency threshold to 0.7.

Specific operation: in fsQCA3.0 software "Analyze" in the selection of "Truth Table Algorithm", in the pop-up dialog box "Variables In the pop-up dialog box "Variables" and "outcome", select the corresponding condition variables and outcome variables, and check "Show solution cas", then we filter the resulting truth table. In "Edit", select "Delete and code", in the "Delete rows width number" box, enter 1, in the "and set outcome variable to 1 for row" box, enter 0.8, if the PRI value for a row is less than 0.7, then manual change the Y value for that row to "0", we obtained the truth Table 7. Where "Number" indicates the number of study cases that meet the combination of conditions. Raw consistency is the degree of consistency of a subset of the outcome affiliation, PRI consist, which refers to the primary condition in raw consistency for each causal condition combination in QCA. In QCA, a primary condition is one that has a high degree of interpretability or importance for the outcome variable. PRI consist denotes the primary condition in raw consistency.

**Table 7:** Truth table.

V1	V2	V3	V4	V5	V6	Number	Y	Raw consist	PRI consist
1	0	0	1	0	1	1	1	1	1
0	1	0	0	1	1	1	1	1	1
0	1	1	1	1	1	7	1	0.976696	0.89911
1	1	0	1	1	1	7	1	0.946074	0.816216
1	0	1	1	0	1	1	1	0.942857	0.744361
0	0	0	1	1	1	2	0	0.939609	0.663367
0	1	0	1	1	1	3	0	0.923937	0.595238
1	0	1	1	1	1	6	0	0.900875	0.696428
0	0	1	1	1	1	6	0	0.890323	0.62222
1	1	1	1	1	1	15	0	0.841369	0.546667
1	0	0	1	1	1	3	0	0.853448	0.492537

Here, the truth table is constructed to facilitate logical operations in the fsQCA3.0 software to analyze the effect that combinations of condition variables have on the results.

### Conditional grouping analysis

In addition to the necessity of a single conditional variable, QCA assesses the adequacy of a conditional grouping, that is, how each conditional variable affects the outcome variable’s occurrence through its combination. In fsQCA 3.0 software, click "Standard Analyses" on the basis of the truth table, and select "Present or absent" in the dialog box to get three solutions: complex, parsimonious, and intermediate solutions. In general, intermediate solutions are preferred to complex and parsimonious solutions because they cover meaningful "logical remainder" [68]. The core conditions of each grouping are identified by comparing the nesting relationships among intermediate and parsimonious solutions. In other words, the conditions that appear in both the intermediate and parsimonious solutions are the grouping’s core conditions, and those that appear only in the intermediate solution are edge conditions. The collation yields Table 8. As shown in Table 8, there are four combinatined paths for construction smart communities in China. In Table 8, Columns 2–5 indicate possible conditional groupings. Table 8 indicates that individual solutions (groupings) have a high level of consistency, consistency are above the theoretical threshold of 0.8, with a minimum value of 0.946074. Furthermore, the solutions’ consistency is 0.940715, which indicates that 94.1% of test areas present a high level of construction among the cases that satisfy the groupings of these four types of conditional high-level smart community construction, and the solutions’ coverage is 0.770989, which indicates that the groupings can explain 77.09% of the cases having high explanatory power. Therefore, these four grouping paths can be sufficient conditions to achieve a high level of smart community construction.

**Table 8:** Conditional grouping analysis results.

Conditional variable	Configuration 1	Configuration 2	Configuration 3	Configuration 4
V1	●	⊗	●	⊗
V2	⊗	●	●	●
V3		⊗	⊗	●
V4	●	⊗	●	●
V5	⊗	●	●	●
V6	●	●	●	●
Consistency	0.951079	1	0.946074	0.976696
Raw coverage	0.236156	0.141836	0.426224	0.50911
Unique coverage	0.0239371	0.0121471	0.131475	0.214362
Solution consistency: 0.940715 Solution coverage: 0.770989				

Note: the large solid circle (●) indicates the core condition’s existence, whereas a large cross circle (⊗) indicates its absence; the small solid circle (●) indicates the edge condition’s existence, whereas the small cross circle (⊗) indicates its absence. Finally, a blank indicates that the condition may or may not exist.

In the realm of influencing factors shaping smart community construction, economic development assumes a dominant driving role in two paths and a dominant restricting role in two paths. A study conducted in India and China corroborates the significance of economic development in this context [45, 63].

Capital investment assumes a multifaceted role, serving as a constraint in one path while acting as driving force in three others [42, 58]. Notably, in Beijing, China, capital investment emerges as a pivotal, leveraging market dynamics for resource allocation. The promotion of Public-Private Partnership (PPP) models and the engagement of diverse social capital have catalyzed significant advancements in Beijing’s smart community initiatives.

Information infrastructure exhibits a nuanced impact, playing a restrictive role in two paths while driving progress in another, consistent with prior research [14, 43, 52, 53]. From the current situation of smart community construction can also be seen, focusing only on the technical construction projects are often "in form", it is difficult to realize the real "wisdom". Smart technology, interwoven with human and managerial competencies, emerges as a linchpin for sustainable community well-being [51]. The current state of smart community construction highlights that a sole focus on technical projects often results in a superficial "appearance" of wisdom, rather than realizing its true essence.

Community governance and public support systems wield considerable influence, albeit in contrasting manners. Community governance facilitates progress along one path while exerting limiting effects in another. Similarly, public support systems exhibit a dual nature, constraining progres in one path while facilitating advancement along three others [42, 44, 45, 50]. Notably, empirical observations across 42 Chinese urban communities underscore the central role of governmental bodies and social organizations in achieving smart community objectives [60], thereby highlighting the critical importance of community governance in this endeavor.

Smart platforms emerge as central facilitators across all pathways, a finding supported by research from Brazil that underscores the significance of information and communication technologies in bolstering smart governance [47–49]. Notably, social media platforms play a pivotal role in bridging communication gaps with the public, enhancing transparency, and fostering resident participation in governance processes [82]. Furthermore, smart platforms serve as the linchpin for delivering a wide array of services to community residents, encapsulating the essence and functionality of smart communities [9, 13, 21].

### Robustness testing

In QCA, there are several main approaches to robustness testing: (1) adjusting calibration anchors or cross-point anchors; (2) lowering the level of consistency of the PRI, e.g., from 0. 7 to 0. 65; (3) raising the case frequency threshold, e.g., from 1 to 2; (4) replacing the antecedent conditions or proxies for the outcomes; (5) raising the level of consistency of the group analysis, e.g., raising 0. 8 to 0. 85 or 0.9; (6) introducing combinations of conditions in which the outcome does not occur for inverse test; (7) randomly deleting part of the sample [83]. This paper will mainly use three methods to test. First, increase the consistency from 0.8 to 0.85. Second, decrease the PRI from 0.7 to 0.65. Third, delete 12 samples randomly. Test results revealed that the new grouping was basically consistent with original results. According to Schneider, Wagemann [84, 85] and other scholars who have proposed criteria for testing the robustness of QCA results, verified the stability and reliability of research results.

## Results and discussion

Drawing from the fundamental conditions delineated within each grouping, and considering the practical context of smart community development, we designate the identified groupings as follows: Economic development driven, Capital investment driven, Economically and capital investment driven, Capital investment and technologically driven.

### Economic development driven grouping

Configuration 1 is expressed as V1*~V2*V4*~V5*V6, wherein economic development*~capital investment*community governance*~public support system*smart platform. This pathway shows that the effectiveness of smart community construction is closely related to the level of local economic development. This path’s consistency is 0.951079; with an original coverage is 0.236156; and unique coverage of 0.0239371, indicates that this pathway can elucidate approximately 24% of the smart community construction cases. We named it: economic development driven. In areas with faster economic development, there tends to be more resources available for smart community construction, as good economic conditions usually mean more available funding and investment. Additionally, it highlights the complementary functions of community governance and smart platforms. This is not only in line with the evaluation indicators of smart community building constructed by scholars [41, 42, 49, 52], but also validates the results of studies in several developing countries, such as China and India [44, 45, 50, 58], once again illustrating the key role of economic development in smart community building.

A study of smart cities in Brazil shows that economic development can drive smart communities [49]. A study of 10 smart cities in Vietnam shows that smart infrastructure, smart human capital development, smart economy, smart government, and smart environment are the key factors influencing their development [50] The influence of economic development on smart community development is a multifaceted and crucial aspect. As economies progress, the capacity for community investment increases, enabling governments and enterprises to allocate more funds and resources toward smart community initiatives. In economically prosperous regions, residents are more likely to possess financial stability and exhibit a willingness to invest in smart community endeavors, such as acquiring smart home devices and engaging in digital community management.

The cases encompassed within this path include Kuancheng District (Changchun City, Jilin Province) and Jiangbei District (Chongqing City).

As an exemplar of this path, Jiangbei District in Chongqing is part of the fourth batch of national pilot zones for community governance and service innovation. Jiangbei District is one of the main urban areas in Chongqing, with a resident population of 902,800 in 2019 and an urbanization rate of 96.7%. The per capita disposable income of residents in the district for the whole year was 42,886 CNY. Among them, the per capita disposable income of urban permanent residents was 43,718 CNY. Finance, commerce and industry are the three pillar industries of Jiangbei District.

In 2019, Chongqing Municipality, a first-tier city in China, recorded a GDP of 2,360.58 billion CNY, with Jiangbei District contributed 124.01 billion CNY, equivalent to 5.3% of the city’s total GDP, ranked fourth among 38 districts (counties) in Chongqing. With the impetus of economic development, smart community construction thrived within this pilot zone. This study underscores the government’s role in ensuring that self-organizations remain aligned with the broad public interest. Widely perceived as a partner or facilitator in realizing collective societal goals [86], Smart community development facilitates the restructuring of social spaces through the fusion of online and offline realms, empowering residents with bottom-up mobility and autonomy, and fostering the emergence of new forms of public spaces and smart ecosystems [87]. the government issued the Implementation Plan for Cultivating and Developing Community Social Organizations to foter the healthy and orderly growth of such entities. Presently, there are, on average, 13 social organizations per community. Smart community development facilitates the restructuring of social space through the fusion of online and offline realms,, empowering residents with bottom-up mobility and autonomy, and fostering the emergence of new forms of public spaces and smart ecosystems.

Moreover, the district has implemented "face-to-face" offline consultation and "key-to-button" online deliberation, establishing a grassroots consultation framework. Residents can participate in online deliberation through the Chongqing Smart Community platform (Yufu WeChat Community Mini Program), particularly crucial during the COVID-19 pandemic, enabling them to engage in discussions and consultations from the comfort of their homes, anytime and from anywhere [88].

### Capital investment driven grouping

Configuration 2 is expressed as ~V1*V2*~V3*~V4*V5*V6, denoting ~economic development*capital investment*~information infrastructure*~community governance*public support system*smart platform. This path highlights the direct impact of capital investment on the construction effect of smart communities. Even if the level of local economic development is relatively low and the information infrastructure and community governance are relatively weak, better construction results can be achieved if sufficient funds are invested in the smart community project. In this case, the availability of funds is a key factor in promoting the construction of smart communities. Public support systems and smart platforms play a supporting role. Given that capital investment serves as the core condition of this path, we denote it as the capital investment driven grouping.

This pathway demonstrates a consistency of 1, with an original coverage of 0.141836, and unique coverage of 0.0121471; thereby elucidating 14% of smart community construction cases.

Dunn (2002) developed a comprehensive evaluation system for smart communities focusing on effectiveness, efficiency, economy, and equity [56], in this assessment system, financial inputs are an important indicator. Another study identified factors affecting smart community in Beijing, including community hardware, network facilities, system platforms, investment funds, and community residents’ awareness levels [58].

The construction of smart communities entails significant costs, necessitating substantial capital support for success. Capital investment serves as a crucial catalyst for smart community construction, with government investment and social engagement being key to achieving capital diversification. Smart community construction entails diverse requirements, including infrastructure development, application of information technology, and procurement of smart equipment. Addressing these needs demands not only substantial financial backing but also concerted efforts from all stakeholders to synergize their contributions.

According to the issued by the Ministry of Housing and Urban-Rural Development Smart Community Construction Guidelines (Trial), the realization of smart community projects necessitates the collective input and support of various stakeholders, including government bodies, enterprises, and community residents. Among these stakeholders, the government, as the primary entity, assumes a critical role in providing financial resources and policy backing.

The sole case encompassed within this path is Qilin District (Qujing City, Yunnan Province).

Qilin District as part of the third batch of national pilot zones for community governance and service innovation, has has demonstrated notable achievements through its strategic enhancements capital investment and information infrastructure. In terms of capital investment, Qujing City’s expenditure on urban and rural community affairs witnessed a substantial rise from 1,709.16 million CNY in 2015 to 2,267.44 million CNY in 2016, indicative of a clear upward trend. Moreover, the Qilin district government allocated over 25 million CNY to optimize its management information system.

Regarding public support systems, Qilin District embarked on comprehensive social governance system reform, delineating the grid into neighborhoods and outlets into groups of residents. This model innovative model organizes communities into grids and residential groups into outlets, staffed by 3,688 grid members(A Grid mamber, or Community Gridman, is a Staff with specific tasks in a community-based grid management organization in a community) Adhering to the "1+5+N" (comprising 1 grid leader, 5 types of grid workers, and N dynamic grid workers), this novel approach significantly bolsters community governance and services.

The advancement of smart communities aims to streamline government affairs while facilitating access to information and promoting inclusive participation [89]. In alignment with smart platform construction, Qilin District not only developed the "Happy Qilin" mobile application but also ensured free WiFi coverage in 32 communities, offering services through the WeChat public number platform.

Pioneering in Yunnan Province, the district established the "Qilin Community Online" website platform, encompassing streets and communities in the central city, along with one network center, eight network stations, 59 network points, and 22 functional modules. These modules span seven major functions, including urban party building, grid management, social services, honest business circle, and big data application, fostering a multi-level application approach ("district, street, community, and grid") that robustly supports smart community construction. Through interconnected devices and smart sensors, real-time community and resident information is gathered and analyzed with big data, facilitating anticipatory governance responses tailored to community needs [13].

### Economically and capital investment driven grouping

Configuration 3 is expressed as V1*V2*~V3*V4*V5*V6, signifying economic development*capital investment*~information infrastructure*community governance*public support system*smart platform. The pathway underscore economic development and capital investment are its core conditions. We named it: economically and capital investment driven. This may mean that in more economically developed regions, in addition to having the advantage of higher economic development, they are also better able to capitalize on the advantage of more abundant funding to support the construction of smart communities. With a consistency of 0.946074, an original coverage of 0.4262248, and unique coverage of 0.131475; this pathway offers explanatory power for 43% of research cases. These findings align with previous studies [14, 50, 53, 55]. For instance, a study examining 10 smart cities in Vietnam identified smart infrastructure, human capital development, smart economy, smart governance, and smart environment as pivotal factors influencing their development [50]. Niaros et al (2017) proposed a multidimensional and multilevel study encompassing government-level support, socio-economic needs, dimensions of the built environment, and levels of connected transportation [55]. This highlights the need for smart community consstrution to be supported by a good economic development and with capital investment from the government.

Specifically, this pathway encompasses the following cases: Haicang District (Xiamen City, Fujian Province); Hangzhou City (Zhejiang Province); Chaoyang District (Beijing City); Shahekou District (Dalian City, Liaoning Province); Xuhui District (Shanghai City); Xicheng District (Beijing City); and Haidian District (Beijing City).

Chaoyang District, a pivotal district within Beijing, was designated as part of the second batch of national pilot zones for community governance and service innovation from 2014 and 2016. Renowned for its robust economic development, the district boasts one of the highest economic indicators nationwide. In 2016, Chaoyang District’s GDP amounted to 5,510.62 billion CNY, approximately constituting 20% of Beijing’s GDP. Notably, its per capita GDP stood at 141,242 CNY, surpassing Beijing’s average by 114690 CNY. Remarkably, this figure dwarfs the national per capita GDP of 54,132 CNY, merely 38% of the GDP of Chaoyang District. Leveraging its economic prowess, Chaoyang District established a dedicated fund to propel pilot zone construction, amassing a cumulative investment of 366.8 million CNY. Presently, smart community construction initiatives in many cities typically follow either a top-down approach driven by local government’s social governance innovation or are spearheaded by real estate, property, and technology enterprises, aiming at technological innovation, market expansion, technology dissemination, and commercial services [90]. In contrast, Chaoyang District has pioneered the establishment of a multi-level, multi-channel, multi-form, mechanism for fund procurement and input assurance. Smart community development demands rationalized institutional mechanisms and exploration of efficient and adaptable governance [91]. The district advances community governance in alignment with Beijing Municipalit’s social governance reform guidelines, fostering street and community management system reform; and fortifying its grid-based social service management system reforms, and fortifying its grid-based social service management as per the "three network integration, two-level closed loop, one-network overall planning" framework, alonside establishing a political discussion mechanism whin the party–government group [65].

To bolster its public support system, Chaoyang District formulated several policies, including the Implementation Plan for the National Pilot Zone for Community Governance and Service Innovation in Chaoyang District, Beijing (2014–2016), and Guiding Opinions on Coordinating and Promoting the Work of the Party, Government, and Groups in Consultation and Co-Governance (for Trial Implementation) [65]. Grid-based social service management system construction of "integration of three networks, two levels of closure, and coordination of one grid."

In terms of enabling smart platform construction, enhancements were made to the community public service comprehensive information platform, alongside the establishment of 233 smart communities following the optimization of centralized business regulations. Chaoyang District implemented one-stop acceptance, full population coverage, full-caliber integration, and full-region office strategies. Leveraging local economic development strength, the district fortified management safeguards, propelled smart community construction, and bolstered smart community development efficacy through synergistic action across three elements [65]. These endeavors, leveraging local economic strength, augmented capital investment, strengthened community governance and public support systems, and improved smart platforms; significantly impacted the outcomes of smart community development.

### Capital investment and technologically driven grouping

Configuration 4 is expressed as ~V1*V2*V3*V4*V5*V6, representing ~economic development*capital investment*information infrastructure*community governance*public support system*smart platform. Capital investment and information infrastructure emerge as the core conditions within this pathway, assuming dominant driving roles, while community governance, public support system, and smart platform serve complementary functions. We named it: capital investment and technologically driven. This pathway emphasizes the joint role of capital investment and information infrastructure. In this case, in addition to sufficient capital investment, the construction and application of information infrastructure is also very important. This means that capital investment and information infrastructure are mutually reinforcing and work together to promote the effectiveness of smart community construction. With a consistency of 0.976696, an original coverage of 0.50911, and a unique coverage of 0.214362; this pathway provides an explanatory framework for 51% of research cases, making it the primary combination pathway for smart community construction.

In recent years, the Chinese government has significantly increased investment in smart community construction and accelerated the development of information infrastructure. China has notably achieved remarkable milestones, boasting the world’s largest fiber optic and mobile broadband network. By the end of 2022, the country had deployed over 2.312 million 5G base stations, accounting for more than 60% of the global total. Moreover, China has achieved high levels of mobile phone and fixed broadband access penetration rates, surpassing global averages [92].

These findings resonate with existing literature [44, 47, 57, 58, 93, 94], which underscores the transformative role of technology, governance, and economic development in smart community construction. Scholars have redefined the concept of smart communities as as human-centric entities empowered by technology to access information and services that inform decision-making processes. Enabling technologies such as cloud computing, crowdsourcing, Big Data analytics, and Internet of Things (IoT) ecosystems, are emphasized in this context [95]. Similarly, studies conducted in Brazil’s major smart cities, including Rio de Janeiro, Porto Alegre, and Belo Horizonte, highlight the critical role of information and communications technology (ICT) in enhancing governance effectiveness and stakeholder integration [48]. Additionally, some scholars advocate for the recognition of institutional infrastructure, physical infrastructure, social infrastructure, and economic infrastructure as the four pillars/themes of smart cities, with the quality of ICT infrastructure significantly influencing smart city performance [96]. In India, widespread Internet availability and mobile phone usage are pivotal in fostering smart communities [45], while studies in Vietnam emphasize the importance of smart infrastructure, human capital development, and smart governance in driving smart city initiatives [50].

This pathway encompasses the following cases: Baita District (Liaoyang City, Liaoning Province); Jianxi District (Luoyang City, Henan Province); Erlianhot City (Inner Mongolia Autonomous Region); Panlong Distric, (Kunming City, Yunnan Province); Yunlong District (Xuzhou City, Jiangsu Province); Taishan District (Tai’an City, Shandong Province); and Xixiu District (Anshun City, Guizhou Province).

As an illustrative case, we examine Baita District (Liaoyang City, Liaoning Province), designated by the Ministry of Civil Affairs (MCA) in September 2012 as one of the national pilot zones for community governance and service innovation. Situated as the central urban area, and the political, economic, cultural, and commercial hub of Liaoyang City, Baita District allocated 240 million CNY, towards bolstering its infrastructure during the pilot phase (2012–2014) to advance smart community development. Research suggests that devolving the provision of public good to the grassroots level is an effective governance strategy [97]. Accordingly, Baita District took a pioneering stance by integrating a network and communications technology platform named "96886" service call platform for residents. This initiative establishished a service model at the governmental level to facilitate social engagement and provide including information service, public security management, healthcare information services, community construction and management, and support for specialized social work management, thereby offering residents diverse community services spanning culture and education, social security, health care, and convenience and benefits [21].

Throughout the smart community construction process, the government assumes the role of shaping, guiding, ensuring, and overseeing the institutional environment [93]. Furthermore, Baita District implemented a series of systems, such as the Opinions on Further Strengthening Community Construction and Governance, to establish a mutually reinforcing institutional framework. The district introduced a community grid management information system based on big data, fostered institutional reforms, established a dedicated community affairs bureau directly directly responsible for community matters, and structured itself into 11 large grids (streets), 63 sub-grids (communities), and 758 grid units (buildings and courtyards). Additionally, it instituted a "one grid, seven members, comprehensive performance" system within the grids [65]. The combined impact of these innovative initiatives has propelled Baita District to achieve notable strides in smart community construction.

In summary, economic development, capital investment, and information infrastructure play a leading role in these four paths, and smart platforms play a complementary role in all of them. They are linked in that these factors may interact with each other and jointly influence the effect of smart community construction. For example, economic development may provide more support for capital investment, while capital investment and information infrastructure construction may also promote each other and jointly promote the construction of smart communities. It is worth stating that the third and fourth path is currently the main path for smart community construction in China, and it also highlights the fact that smart community construction is a systematic project that is affected by a combination of factors.

## Conclusion

The endeavor to construct smart cities is pervasive across China, with over 89% of cities above the sub-provincial level, and 47% of cities at the county level and above have proposed to build smart cities [98]. Within the global context of smart city evolution, the construction of smart community emerges as a critical facet, strategically positioned to foster innovation in community governance models, bolster urban modernization efforts [99], and cultivate fresh urban competitive advantages. By scrutinizing 52 of national pilot zones for community governance and service innovation in China, this study has crafted an analytical framework elucidating pivotal influencing factors, include economic development, capital investment, information infrastructure, community governance, public support system and smart platform. Employing QCA as the methodological cornerstone, our inquiry has delineated four viable pathways conducive to the realization of smart community construction.

This research not only introduces novel methodologies and case studies to the discourse surrounding smart community construction but also advances our comprehension of the underlying mechanisms steering this domain. Furthermore, it lays the groundwork for a more comprehensive and systematic theoretical framework, furnishing theoretical guidance crucial for the effective realization of smart community initiatives. At the same time, it provides scientific basis for government departments to formulate relevant policies. Especially for other developing countries, these policy recommendations may have strong reference value and help them achieve better results in smart community construction. Relevant institutions and communities can optimize the allocation of resources and improve governance mechanisms in a targeted manner, thus enhancing the efficiency and quality of smart community consruction.

The following conclusions were drawn from this study:

First, it is evident that smart community construction is significantly influenced by community governance, public support systems, and smart platforms (See Table 6), these three conditional variables indicate necessary conditions for smart community construction.

Scholars such as Caragliu et al. (2009) emphasize the transformative potential of smart communities, highlighting the importance of efficient governance structures in fostering citizen engagement and participatory decision-making processes [4]. Batty(2013) noted that smart community is a change in the way communities, residents and governments interact, and a governance model that makes community governance more effective [10]. A study of German and Chinese cities found that the strong role of states and municipal governments in Germany, or the top-down style and the omnipresent presence of the national political machinery in China [100]. For example, community associations, nonprofit organizations, and ad-hoc task groups [32] play active roles in smart communities. Effective community governance helps improve overall community management by promoting participatory governance and facilitating the use of efficient operating systems. The integration of smart platforms, characterized by advanced technological infrastructures, profoundly influences residents’ urban experiences [101]. These platforms facilitate efficient service delivery, enabling access to community data and enhancing public service provision. Consequently, the development of smart platforms emerges as a requisite strategy for realizing the full potential of smart community initiatives. Moreover, it is imperative to recognize that the advancement of smart communities transcends mere technological innovation. While digital infrastructure and smart technologies are indispensable enablers, the essence of smart community development lies in the creation of inclusive and socially cohesive environments, the cultivation of public spirit is the core of the smart community [102]. As noted by Komninos (2002), smart communities should prioritize the cultivation of public spaces that foster social interaction, collective problem-solving, and the exchange of ideas [103]. In this context, the construction of robust public support system assumes paramount importance. This includes the formulation of supportive policies, the establishment of agile organizational structures, and the cultivation of skilled human capital [104]. Strong public support systems, as argued by Schaffers (2011), are essential for overcoming institutional barriers and fostering collaboration among diverse stakeholders within smart communities [105].

Second, four combinatorial paths were derived from the analysis: economic development driven, capital investment driven, economically and capital investment driven, capital investment and technologically driven. Among these paths, economic development, capital investment, and information infrastructure emerge as pivotal drivers of smart community construction. Concurrently, the role of smart platform emerges as complementary across all pathways, underscoring the importance of a fully functional smart platform within smart communities (See Table 8). The result is consistent with the research findings of Liang Li, Eliasbibri and other scholars [42, 44, 58] that is, the levels of economic development, capital investment, and information infrastructure directly affect the effectiveness of smart community construction. In the four parameterization paths of smart community construction, economic development plays a key role in the two paths parameterizations 1 and 3. Capital investment plays a leading role in the paths configurations 2, 3, and 4, whereas information infrastructure leads in the path parameterization 4. Therefore, economic development, capital investment, and information infrastructure are important conditional factors influencing the effectiveness of smart community construction. By understanding and prioritizing these factors, stakeholders can navigate the complexities of smart community development, ultimately fostering resilient and technologically empowered communities.

Third, the identification of the four critical paths in this study presents promising for smart community construction, collectively covering 77.1% of cases analyzed (See Table 8). This comprehensive coverage suggests applicability across a majority of smart community scenarios, encompassing both the types lacking all types of conditions and paths where multiple conditional elements perform well. In practical terms, the delineation of these paths offers valuable guidance for stakeholders involved in smart community initiatives. It underscores the importance of selecting a construction path tailored to the unique characteristics and circumstances of each pilot zone. Notably, the third and fourth path emerges as a prevalent choice among current smart community construction endeavors in China. Once again, it has been verified that the construction of smart communities is a complex systematic project, and its effect is comprehensively affected by multiple factors.

Based on our research results, we propose the following strategies: First, Enhance innovative techniques in community governance by Leveraging party organizations’ resource integration capabilities and promoting collaborative governance among social organizations and residents. This fosters a diverse social space, empowering residents as primary governance actors and facilitating high-quality smart community development [87]. Strengthen the construction of public support systems by improving top-level institutional design, scientifically planning construction systems, and ensuring operational mechanisms for smart communities. This includes reforming grassroots governance and smart grid management. Second, strengthen the construction of public support system.by improving top-level institutional design, scientifically planning construction system, and ensuring operational mechanisms for smart communities. This includes reforming grassroots governance and smart grid management [106]. Improve information infrastructure and smart platforms in communities through increased financial investment from governments. Leveraging platforms like WeChat mini programs can provide residents with convenient services and opportunities for governmental participation, while digital governance platforms can address residents’ concerns promptly. Third, improve information infrastructure and smart platforms in communities through financial investment from governments. Leveraging platforms like WeChat mini programs can provide residents with convenient services and opportunities for governmental participation, while digital governance platform can address residents’ concerns promptly. Fourth, promote local economic development and capital investment by fostering cooperation between the government and social capital [107, 108]. Exploring market-based governance models and encouraging enterprises to provide services can driven community development [109]. In conclusion, smart community construction should consider holistic, systematic, and specialized, prioritizing residents’ well-being and adapting strategies to local local contexts and resources.

This study has the following limitations: Smart community construction is a dynamic process and influencing factors may change over time. As a result, the timeframe in the study may not cover a long enough period of time to reflect trends in influencing factors over time. In addition, this study selected six influencing factors to start the analysis, which, may be many have neglected other factors affecting the construction of smart community.

Future studies could conduct comparative analyses of smart community development in different regions or countries to reveal differences in influencing factors in different contexts and provide a more comprehensive understanding. Long-term tracking studies could also be conducted to understand the trends and long-term effects of the influencing factors.

## Supporting information

S1 Datasheet DOI:https://doi.org/10.6084/m9.figshare.25380343.v1. (XLS)
